# Navigating end-of-life decision-making on ECLS discontinuation in children: insights from the international ENSURE survey

**DOI:** 10.1007/s00431-025-06629-0

**Published:** 2025-12-02

**Authors:** Angelo Polito, Marcel Fleig, Janet Kelly-Geyer, Aparna Hoskote, Heidi Dalton, Joe Brierley, Ravi R. Thiagarajan, Alexander Supady, Justyna Swol

**Affiliations:** 1 Paediatric Intensive Care Unit, Department of Paediatrics, Gynaecology and Obstetrics, Geneva University Hospital, Geneva University of Medicine, Geneva, Switzerland; 2 Department of Respiratory Medicine, Paracelsus Medical University, Nuremberg, Germany; 3 Department of Intensive Care and Neonatology and Children’s Research Centre, University Children’s Hospital Zurich, University of Zurich, Zurich, Switzerland; 4 Pediatric Cardiac Intensive Care Unit, Great Ormond Street Hospital for Children NHS Foundation Trust, London, UK; 5 Professor of Surgery and Pediatrics, Creighton University, Omaha, NE USA; 6 Paediatric Bioethics Centre, NIHR Great Ormond Street Hospital Biomedical Research Centre, London, UK; 7 Department of Cardiology, Boston Children’s Hospital, Boston, MA USA; 8 Department of Pediatrics, Boston Children’s Hospital, Boston, MA USA; 9 Interdisciplinary Medical Intensive Care, University Medical Center, Faculty of Medicine, University of Freiburg, Freiburg, Germany; 10 Department of Cardio-Thoracic Surgery, Cardiac Surgery and Extracorporeal Life Support, ECLS Program, Cardiovascular Research Institute Maastricht (CARIM), Heart & Vascular Centre MUMC+, Maastricht, Netherlands

**Keywords:** ECLS, PICU, End-of-life care, Ethical decision-making, Children

## Abstract

Healthcare professionals face significant challenges when deciding whether to continue or withdraw Extracorporeal Life Support (ECLS) in pediatric patients, which may cause moral distress. The ENd-of-life deciSion-making dURing ECLS (ENSURE) survey investigates the perspectives of European healthcare professionals on ECLS discontinuation in pediatric patients considering the potential impact of cultural, religious, and regional factors. A 23-item mixed-method questionnaire survey was conducted across 21 European countries in different languages, assessing respondent demographics, ethical viewpoints, and institutional practices. The analysis focused on differences in decision-making based on geographic region, profession, and religion. A total of 192 healthcare professionals participated, with 68% from ELSO centers. Most respondents (77%) agreed that ethics consultations would benefit patients and families when ECLS fails to meet treatment goals. Preferences for discontinuing ECLS varied regionally, with practitioners from Northern and Western Europe more likely to favor the discontinuation of ECLS with comfort care (62–68%) compared to those from Southern and Eastern Europe (34–50%). Religious beliefs might influence decisions, with Christian and agnostic/atheist participants more likely to support ECLS discontinuation.

*Conclusion: *Decision-making surrounding ECLS discontinuation in pediatric patients is influenced by regional, cultural, and possibly religious factors. There is a need for standardized guidelines and expanded access to ethics consultations to support healthcare professionals and reduce moral distress in these complex cases. Addressing discordance in clinical teams and enhancing palliative care access may improve the decision-making process and patient care outcomes.

**Table:** 

**What is Known:** •*Decisions about ECLS withdrawal in pediatric patients are ordinarily made in close consultation with families, who are particularly influenced by emotional factors.* •*There is a scarcity of literature related to the ethics of ECLS initiation, continuation, and termination in children, providing minimal, specific ethical recommendations.*	
**What is New:** •*The ENSURE study evaluates current viewpoints of ECLS professionals across Europe and investigates influencing respondent or institutional characteristics.* •*Guidelines for managing ECLS patients considering regional, religious, and cultural factors could reduce variability in treatment and mental distress in decision-making.*

**What is Known:**

•*Decisions about ECLS withdrawal in pediatric patients are ordinarily made in close consultation with families, who are particularly influenced by emotional factors.*

•*There is a scarcity of literature related to the ethics of ECLS initiation, continuation, and termination in children, providing minimal, specific ethical recommendations.*

**What is New:**

•*The ENSURE study evaluates current viewpoints of ECLS professionals across Europe and investigates influencing respondent or institutional characteristics.*

•*Guidelines for managing ECLS patients considering regional, religious, and cultural factors could reduce variability in treatment and mental distress in decision-making.*

## Introduction

The significant increase in pediatric Extracorporeal Life Support (ECLS) utilization over time in recent years has prompted important challenges regarding decision-making with regard to candidacy or continuation of ECLS in the face of poor prognosis or lack of an exit strategy [1, 2]. In fact, there may be situations where patients relying on ECLS support cannot be bridged to recovery, transplant, or another device as a destination therapy, yet they can continue to survive with ongoing ECLS assistance [3]. Due to the uncertainties in predicting outcomes, difficult decisions about ECLS discontinuation may emerge, posing significant challenges for healthcare professionals and exposing them to moral distress and burnout [4, 5]. Additionally, the primary role parents play in collaborative decision-making for PICU patients leads to a significant overlap between the interests of the child and those of the family, introducing additional complexities to end-of-life care decisions for children on ECLS [6]. Furthermore, cultural and religious backgrounds of families and healthcare professionals compound additional complexity related to end-of-life decisions [7]. Even within regions with cultural homogeneity, significant variation in end-of-life viewpoints among individual ECLS practitioners within the same ICU has been noted [8].

There is a scarcity of high-quality literature related to ECLS initiation, continuation, and termination in children, providing only minimal, specific recommendations [9]. Enhanced comprehension of ECLS providers’ perspectives surrounding the discontinuation of ECLS in children could result in better care and potentially alleviate the moral distress experienced by healthcare professionals navigating this complex clinical decision. Our objective was to evaluate the current viewpoints of healthcare professionals across Europe who are engaged in providing ECLS care. Specifically, we aimed to understand clinicians’ perspectives and attitudes on challenges related to withdrawing ECLS in children. Furthermore, we aimed to investigate whether respondent or institutional characteristics influenced these viewpoints.

## Materials and methods

### Survey design

The Extracorporeal Life Support Organization (ELSO) is an international nonprofit consortium that provides education, guidelines, and data support for the use of ECLS to treat critically ill patients [10]. The ENd-of-life deciSion-making dURing ECLS (ENSURE) survey questionnaire was developed by a EuroELSO research group through an iterative consensus process based on the CHERRIES statement [11]. Details regarding the survey development and promotion are found in the supplementary material.

The questionnaire was composed of three sections: The first dealt with general questions on hospital details, ICU profile, ECLS provision, and participants’ profession. The second part was designed to elicit clinical questions, institutional protocols, management, and palliative care provision, including questions about respondents’ actual practices when ECLS goals are no longer achievable. The final section included three optional, scenario-based questions for healthcare professionals involved in pediatrics. The purpose of each scenario was to examine the factors affecting the decision to continue or discontinue ECLS, considering the degree of cardiopulmonary compromise and related comorbidities [12]. We analyzed factors influencing this choice, including medical history, comorbidities, and context. For each scenario, we asked whether stopping ECLS alone or with life-sustaining treatments (LST) such as mechanical ventilation and vasopressor administration was the best course. We explored how geographic location, religion, and profession related to these decisions and assessed the impact of discordance among providers, parents/guardians, and between them on decision-making.

European regions were defined by the UN Geoscheme for Europe [13]. No patient data was collected or stored, complying with GDPR. The EuroELSO Review Board deemed the study exempt as subjects could not be identified.

### Statistical analysis

Results are presented as numbers and percentages to summarize responses and characteristics of participants and their institutions. Aggregated data were regarded as non-parametric presented as median (interquartile range (IQR) = 25–75%). Results from multiple-choice questions are expressed in median, participants’ extent of agreement or disagreement in mean, or standard deviation. No inferential statistics were conducted due to the nature of the data collected and the study’s design.

For regional analysis, participants were allocated to Eastern Europe, Northern Europe, Southern Europe, or Western Europe according to the United Nations Geoscheme for Europe, created by the United Nations Statistics Division (UNSD) [19, 20]. Israel, Turkey, and Kazakhstan were included separately in the analysis of European countries and added to the descriptive statistics. All analyses were performed using R-4.2.2 software (R Foundation for Statistical Computing, Vienna, Austria; URL https://www.R-project.org).

## Results

### Participants and institutional characteristics

Of 192 healthcare professionals from 21 European countries, the highest representation came from Switzerland (44, 23%), the UK (18, 9%), France (17, 9%), Israel (17, 9%), and the Netherlands (17, 9%). Most (131, 68%) worked at Extracorporeal Life Support Organization (ELSO) centers, offering cardiac ECLS (90%), respiratory ECLS (89%), and extracorporeal cardiopulmonary resuscitation (ECPR) (74%). Respondents primarily managed adult, pediatric, and neonatal patients (101, 53%), with 74 (39%) focusing on pediatric and neonatal cases. Physicians comprised 119 (62%) respondents, followed by nurses (48, 25%), ECLS specialists (16, 8%), and perfusionists (9, 5%) (Table 1).

**Table 1:** Participants’ characteristics (Merged Christians included Protestant, Catholic, and Orthodox, and merged Others included Jehovah’s Witnesses, Muslims, Jews, Hindus, and Buddhists.)

**Region (*N* = 192)**	***n* (%)**
Western Europe	90 (47)
Southern Europe	44 (23)
Northern Europe	34 (18)
Eastern Europe	24 (12)
**ECLS center characteristics (*N* = 192)**	***n* (%)**
Adults, pediatric and neonatal	101 (53)
Pediatric and neonatal	74 (39)
Pediatric and adult	7 (4)
Pediatric only	4 (2)
Others	6 (3)
**Professional categories (*N* = 192)**	***n* (%)**
Physician	119 (62)
Nurse	48 (25)
ECLS specialist	16 (8)
Perfusionist	9 (5)
**Religion as reported (*N* = 192)**	***n* (%)**
Christian	82 (43)
Agnostic/atheist	59 (31)
Other/unknown	51 (26)

### Ethical and institutional practices

Only 100 respondents (52%) had access to a Clinical Ethics Committee, and 31 (16%) had clinical ethicists available, while 41 (21%) reported no ethics support. Most (147, 77%) indicated that ethics consultations would benefit patients and families when ECLS fails to meet treatment goals, and 155 (81%) noted benefits for healthcare professionals. Only 29 (15%) had institutional protocols for resolving disagreements between physicians and families regarding LST restrictions.

When ECLS failed, 88 respondents (50%) favored discontinuing ECLS and LST while providing comfort care, 33 (19%) chose no treatment escalation while continuing ECLS, 22 (12%) opted to discontinue ECLS with LST continuation, and 5 (3%) maintained current management. Preferences varied by region: Northern and Western Europe favored ECLS discontinuation with comfort care (23, 68% and 56, 62%, respectively), while Southern and Eastern Europe preferred ECLS continuation with limiting LST escalation (10, 34% and 12, 50%, respectively). Religious beliefs influenced choices, with Christian and agnostic/atheist respondents more likely to support ECLS discontinuation and providing comfort care (Table 2).

**Table 2:** Patient management in case ECLS is not achieving treatment goals

	**No change in patient treatment,** *n* (%)	**Continue ECLS, no escalation,** *n* (%)	**Stop ECLS, continue all other LST,** *n* (%)	**Stop ECLS, stop LST, comfort care,** *n* (%)	**I do not know/other,** *n* (%)
**Total (*N* = 177)**	5 (3)	33 (19)	22 (12)	88 (50)	29 (16)
**European region (*N* = 177)**					
Western (*n* = 90)	1 (1)	10 (11)	10 (11)	56 (62)	13 (14)
Southern (*n* = 29)	2 (7)	10 (34)	5 (17)	7 (25)	5 (17)
Northern (*n* = 34)	0 (0)	1 (3)	2 (6)	23 (68)	8 (24)
Eastern (*n* = 24)	2 (8)	12 (50)	5 (21)	2 (8)	3 (6)
**Religion (*N* = 177)**					
Christian (*n* = 82)	1 (1)	13 (16)	14 (17)	38 (46)	16 (20)
Agnostic/atheist (*n* = 59)	1 (2)	11 (19)	5 (8)	36 (61)	6 (10)
Other/unknown (*n* = 36)	3 (8)	9 (25)	3 (8)	14 (39)	7 (20)

Vasoactive drugs (129, 67%), continuous renal replacement therapy (116, 60%), and mechanical ventilation (112, 58%) were the most common “ceiling of care” treatments. In Southern and Eastern regions, 13 (30%) and 7 (29%) respondents chose “no limitations” compared to 2 (6%) and 8 (9%) in Northern and Western regions *(Suppl. Tab. 1)*.

Palliative care was accessible to 117 respondents (61%), but 89 (46%) noted no patient referrals, with 56 (29%) citing lack of access. Systematic referrals for futile cases occurred among 22 (11%), while 62 (32%) referred on a case-by-case basis *(Suppl. Tab. 2)*. ECLS discontinuation discussions always involved physicians, with nurses (144, 75%) and relatives (111, 58%) frequently included, alongside perfusionists (61, 32%), ethicists (46, 24%), chaplains (42, 22%), and physiotherapists (27, 14%) *(Suppl. Tab. 3)*. Donation after circulatory determination of death (DCD) programs was available to 98 (51%) respondents.

### Case scenarios

#### Case 1: 6-year-old with viral myocarditis

**Fig. 1:**
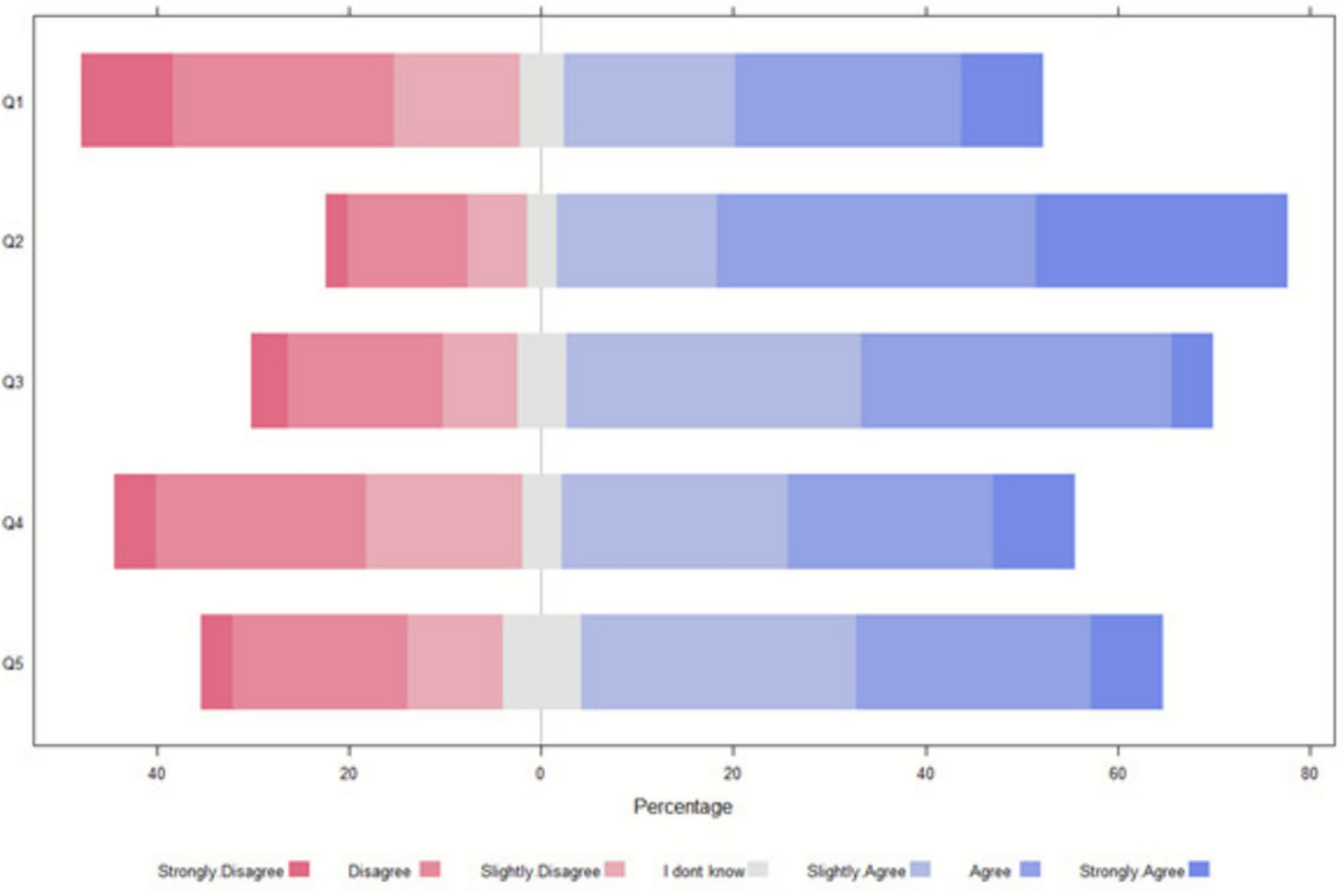
Regarding case 1, what is the most appropriate clinical decision and what factors influenced the decision (*N* = 192). Legends: Q1: Stop ECLS (without limitation of LST). Q2: Stop ECLS (with limitation of LST) and reorientation to comfort care. Q3: Disagreement among healthcare providers might have an impact on my decision. Q4: Disagreement between the parents/guardians might have an impact on my decision. Q5: Disagreement among healthcare providers and parents/guardians might have an impact on my decision

In case 1, 136 respondents (76%) favored discontinuing ECLS and withholding LST, citing quality of life concerns and the futility of continued LST due to hypoxic encephalopathy (Fig. 1). Support for discontinuation was stronger in Western (81, 90%) and Northern (29, 85%) regions compared to Southern (23, 52%) and Eastern (13, 54%) regions, where preferences were more diverse. Christian and agnostic/atheist respondents also strongly supported discontinuation (66, 80% and 52, 88%).

#### Case 2: 3-month-old with PARDS

**Fig. 2:**
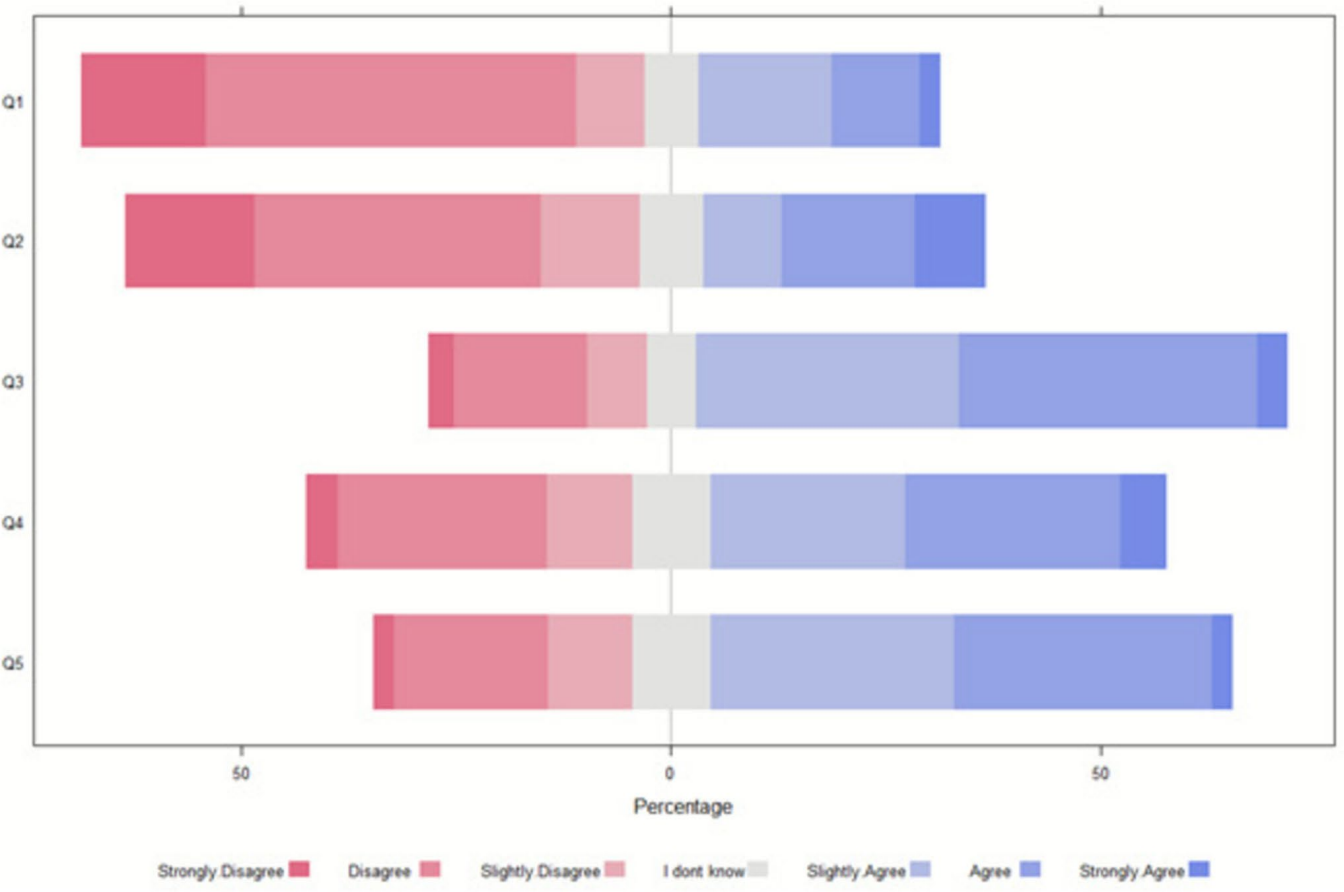
Regarding case 2, what is the most appropriate clinical decision and what factors influenced the decision (*N* = 174). Legends: Q1: Stop ECLS (without limitation of LST). Q2: Stop ECLS (with limitation of LST) and reorientation to comfort care. Q3: Disagreement among healthcare providers might have an impact on my decision. Q4: Disagreement between the parents/guardians might have an impact on my decision. Q5: Disagreement among healthcare providers and parents/guardians might have an impact on my decision

In case 2, 104 respondents (59%) opposed discontinuing ECLS with continued LST, and 114 (65%) opposed discontinuation while withholding LST, primarily due to concerns that insufficient time had elapsed for lung recovery despite complications (Fig. 2). Opposition was higher in Southern (31, 76%) and Eastern (17, 81%) regions compared to Western (42, 51%) and Northern (14, 47%) regions. Non-Christian/agnostic respondents opposed discontinuation more frequently (23, 74%).

#### Case 3: 3-year-old with trisomy 21

**Fig. 3:**
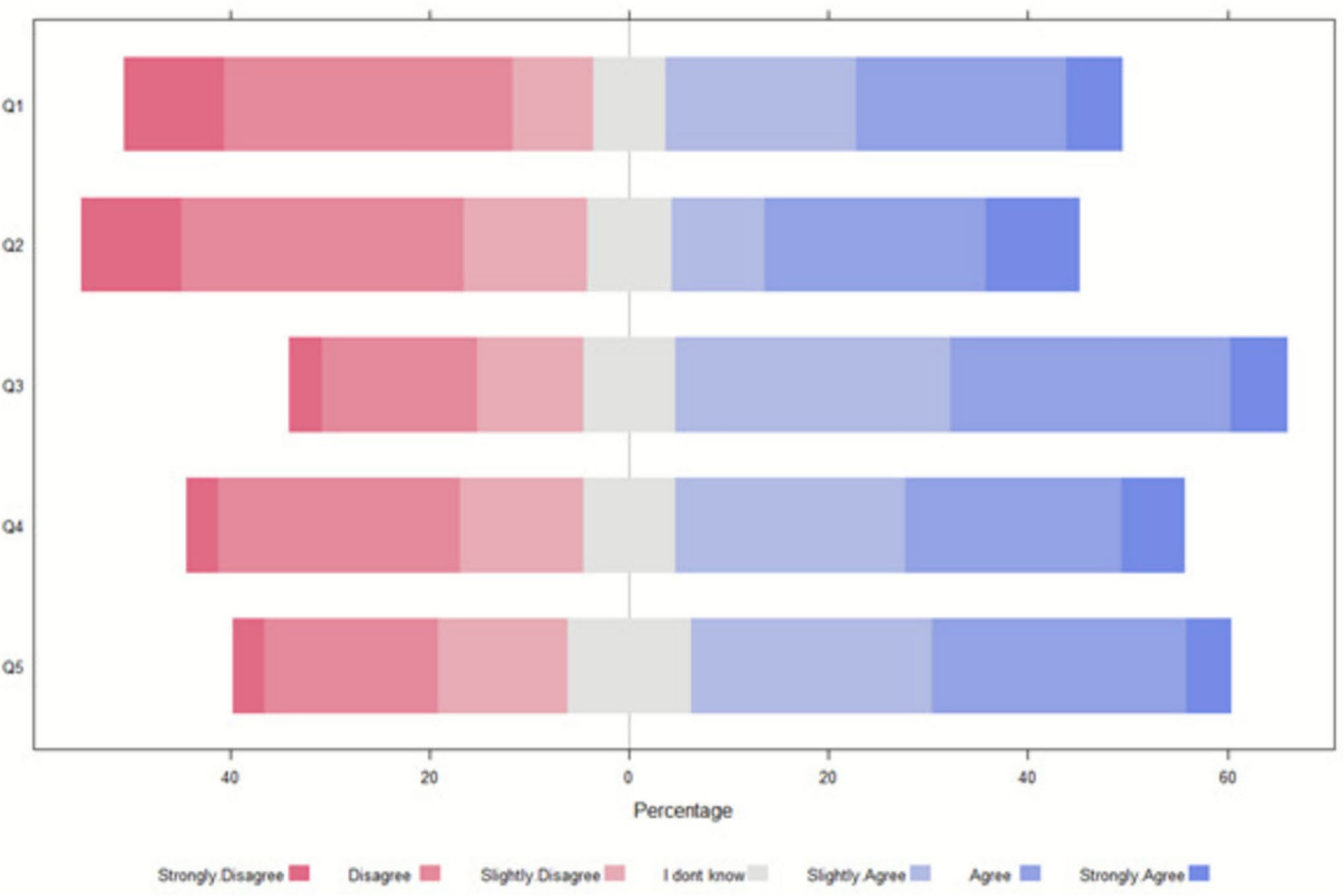
Regarding case 3, what is the most appropriate clinical decision and what factors influenced the decision (*N* = 162). Legends: Q1: Stop ECLS (without limitation of LST). Q2: Stop ECLS (with limitation of LST) and reorientation to comfort care. Q3: Disagreement among healthcare providers might have an impact on my decision. Q4: Disagreement between the parents/guardians might have an impact on my decision. Q5: Disagreement among healthcare providers and parents/guardians might have an impact on my decision

In case 3, respondents were evenly split: 82 (50%) opposed and 66 (40%) favored ECLS discontinuation with LST limitation, while 76 (47%) opposed and 74 (46%) favored discontinuation without LST limitation (Fig. 3). Reasons for discontinuation included futility due to multi-organ failure, while others supported continuation to allow time for transplant eligibility.

Disagreements among providers, parents and families, and between these groups significantly influenced decisions in all scenarios (Figs. 1, 2, and 3). Respondent profession did not notably affect preferences.

## Discussion

### Differences in decision-making

Our survey of 192 healthcare professionals across Europe revealed that most favored discontinuing ECLS and providing comfort care when treatment goals were unmet, with preferences varying by region and religious affiliation. A patient-centered model emphasizing autonomy and family involvement is increasingly prominent in Europe [14].

However, regional variations persist, with Western and Northern Europe favoring ECLS and LST discontinuation, while Southern and Eastern Europe lean toward treatment continuation [15, 16]. These differences likely reflect cultural, religious, and case-mix variations, as well as disparities in end-of-life legislation and societal attitudes [7].

In Western tradition, discontinuation and withholding of LST are ethically equivalent, yet discontinuation poses unique emotional and logistical challenges [17]. Some perceive it as a choice between preserving life and allowing death [8]. A no-escalation strategy offers a potential solution. Our findings indicate that many respondents favor non-escalation of treatment, highlighting significant variability in end-of-life practices. Religious beliefs can influence end-of-life decisions in the ICU [18–21]. Although one study found physicians’ religious affiliations are not the main factor in these practices [14], the results suggest that religion may play a role in certain cases. Our findings indicate that Christian and agnostic/atheist respondents more often de-escalate therapy and redirect to comfort care when ECLS fails. This reflects differing views on life and death boundaries, impacting decisions [22]. The small number of non-Christian/agnostic respondents calls for cautious interpretation and further research.

Integrating ethics consultation into ECLS care is crucial when treatment goals are unmet. Ethics consultations are less common in pediatric ECLS than in adult ECLS [23], primarily addressing ECLS discontinuation. Most respondents agreed that ethics consultations benefit patients, families, and healthcare professionals. Limited access to Clinical Ethics Committees and ethicists highlights the need for better access. While evidence for effectiveness remains limited [24], institutional policies promoting ethics consultation can enhance patient-centered care and shared decision-making, while reducing moral distress. Decision-making is often hindered by disagreements among healthcare providers and families. Institutions may develop policies and protocols to address these challenges, including clear guidelines for managing conflicts.

Open communication and collaboration between healthcare providers and families are crucial. A structured conversation guide for ECLS [5] may alleviate distress for families and clinicians by discussing when ECLS is no longer beneficial, facilitating end-of-life planning, and improving communication and bereavement support. While most respondents had access to palliative care, referrals were inconsistent, with only 32% occurring on a case-by-case basis and only 11% systematically *(Suppl. Tab. 2)*. Although palliative care is recommended for all terminally ill patients [25], standardized guidelines for concurrent referrals are lacking. While higher palliative care visit frequency is associated with prognostic uncertainty, medical complexity, and run duration [26], the impact of palliative care on pediatric outcomes remains unknown [4]. To improve care, access to advanced care planning and timely palliative care upon ECLS discontinuation should be ensured. Future research should focus on developing referral triggers and evaluating the impact of these interventions on outcomes.

### Case scenarios

*Case 1* highlights the impact of neurological injuries on decisions to withdraw ECLS. Neurologic injury is a poor prognostic factor for ECLS patients, contributing to high morbidity and mortality [27]. Less than half of pediatric ECLS survivors have favorable neurologic outcomes at discharge [28]. However, data on neurologic injury’s role in discontinuation decisions is scarce [29]. According to the Society of Critical Care Medicine, care is deemed inappropriate if there is no reasonable expectation of neurologic improvement [30]. Neurologic injuries influence early discontinuation decisions in adult ECPR [31].

Most respondents in this study would withdraw ECLS and discontinue other LST in such circumstances, reflecting the importance clinicians place on neurologic injury and its potential impact on quality of life when making ECLS discontinuation decisions.

*Case 2* involves a patient with severe respiratory and cardiovascular complications. Many respondents opposed ECLS discontinuation, believing the lungs might need more time to heal, consistent with ELSO pediatric respiratory ECLS guidelines, which recommend extended ECLS support for lung recovery [32].

*Case 3* examines a trisomy 21 patient with severe ECLS complications. Historically, ECLS use in trisomy 21 patients was approached with caution due to the increased morbidity and mortality risks [33, 34]. However, studies show similar ECLS mortality rates for trisomy 21 patients and those without genetic conditions [35, 36]. Respondents were divided on continuing or withdrawing ECLS, citing either perceived futility or the need for more time to become transplant candidates. Notably, trisomy 21 was not cited as a reason for ECLS discontinuation, suggesting that it may no longer be considered a significant obstacle for ECLS in children.

There are established definitions of futile and ineffective interventions, along with consensus statements on end-of-life care for critically ill patients [25, 30]. However, these resources are often broad and lack the detail needed for specific patient cases, complicating their application. Developing clear, consistent guidelines for managing ECLS patients in various scenarios could reduce variability in treatment approaches and aid decision-making. These guidelines should consider regional, religious, and cultural factors that influence patient care. Recognizing the role of personal beliefs and values in decision-making processes can help healthcare providers and families navigate complex situations with greater clarity. Future research could explore ways to better understand and incorporate these factors into clinical decision-making, such as developing tools or resources to aid in this process.

### Limitations

This study has several limitations. The lack of a defined sample size prevents estimating the response rate, introducing potential response bias. Due to the survey’s distribution via the EuroELSO mailing list, social media, the EuroELSO website, and the 10th EuroELSO Congress app, we could not track how many people were exposed to the survey. While this limits response rate calculations, broad outreach was made to minimize selection bias and ensure sample representativeness. The survey did not capture institutional affiliations, limiting regional or institutional analysis. We also could not control for multiple submissions due to its anonymous nature. Optional religious background declaration led to inconsistent abstention rates among different groups, limiting the depth of the analysis on cultural or religious influences. Uneven response distribution across professions and regions, particularly in smaller groups, limited robust statistical analysis.

Additionally, while the survey captured respondents’ reported clinical practices, these self-reports may not fully reflect real-world actions due to institutional, legal, or resource constraints not assessed here. Most respondents cared for a mix of patients rather than exclusively pediatrics, which may influence the results, particularly given that prognostication uncertainty is a predominant issue in pediatric care.

The free text comments were few in number and were not categorized using specialized software. Our study aims to explore the healthcare professionals’ perspectives on practical, clinical aspects of ECLS discontinuation. There are individual reports suggesting that decision-making can result in moral distress, so one is led to wonder naturally. An attempt to ascertain if the respondent or their team members had personally experienced moral distress because of their/others’ decision-making was not included in the survey. A full ethical analysis was beyond the scope of the manuscript and would require a different research approach.

The availability and organization of ECLS centers may vary within different European regions [37], which may further influence attitudes toward withdrawal of support. However, owing to the anonymous nature of the respondent answering, this may be challenging to ascertain among respondents. Countries like Switzerland, Germany, the UK, Scandinavia, and some others typically have high ECLS availability, with established ECLS centers, referral systems, and specialized transport teams. These regions often adhere to standardized guidelines and have capacity for prolonged support and multidisciplinary decision-making whereas in other regions, protocols may be less standardized, leaving decisions more influenced by local norms, individual clinicians, or family wishes. These variations suggest that decisions about withdrawal of ECLS support might be shaped not only by cultural and religious factors, but also by structural and economic realities of healthcare delivery in different parts of Europe, which may further influence attitudes toward withdrawal of support.

## Conclusion

The management of ECLS patients facing treatment failure is influenced by factors such as geographical location and disagreements among healthcare providers and families. Referrals to ethics and palliative care services should also be considered to ensure balanced, informed decisions. Future research should expand this and other surveys globally to other ECMO centers and prioritize further observational and qualitative studies, such as interviews or focus groups with diverse healthcare professionals.

## Data Availability

Access to the original survey questions in several languages, the survey results data and the professional data analysis can be provided by the corresponding author via e-mail: marcel.fleig@stud.pmu.ac.at.

## Abbreviations

CHERRIES: Checklist for Reporting Results of Internet E-Surveys
ECLS: Extracorporeal Life Support
ECMO: Extracorporeal Membrane Oxygenation
ECPR: Extracorporeal cardiopulmonary resuscitation
ELSO: Extracorporeal Life Support Organization
ENSURE: ENd-of-life deciSion-making dURing ECLS
EuroELSO: European Chapter of the Extracorporeal Life Support Organization
GDPR: General Data Protection Regulation
ICU: Intensive Care Unit
IQR: Interquartile range
LST: Life-sustaining treatments
PICU: Pediatric Intensive Care Unit
UK: United Kingdom
UN: United Nations
UNSD: United Nations Statistics Division
URL: Uniform Resource Locator
